# Value of pneumococcal PCR in respiratory samples for exclusion of pneumococcal pneumonia

**DOI:** 10.1093/jacamr/dlad115

**Published:** 2023-11-02

**Authors:** Sam Van Goethem, Philippe Van Lint, Philippe Willems, Bruno Van Herendael, Katrien Hoet

**Affiliations:** Laboratory of Medical Microbiology, Vaccine and Infectious Diseases Institute, University of Antwerp, Antwerp, Belgium; Hospital Outbreak Support Team, ZAS Hospital Network, Antwerp, Belgium; Medical Microbiology Laboratory, GZA Hospitals Antwerp, Antwerp, Belgium; Medical Microbiology Laboratory, GZA Hospitals Antwerp, Antwerp, Belgium; Medical Microbiology Laboratory, GZA Hospitals Antwerp, Antwerp, Belgium; Medical Microbiology Laboratory, GZA Hospitals Antwerp, Antwerp, Belgium

## Abstract

**Background:**

*Streptococcus pneumoniae* is the main aetiological agent in bacterial pneumonia. Therefore pneumococcal PCR is often included in respiratory multiplex PCR panels, both commercial and in-house. But respiratory PCR results for *S. pneumoniae* are difficult to interpret due to frequent non-pathogenic colonization on the mucosal surface of the upper airways with pneumococci or to cross-reaction of the PCR target in non-pneumococcal streptococci. In this study we investigated the value of *lytA* gene pneumococcal PCR in patients presenting with pneumonia.

**Objectives:**

To assess the utility of *lytA* gene detection for *S. pneumoniae* in a respiratory multiplex quantitative PCR (qPCR) panel for patients presenting with pneumonia.

**Methods:**

A retrospective study was conducted for *lytA* gene results as target for *S. pneumoniae* in hospitalized patients who were diagnosed with pneumonia and for which a respiratory multiplex PCR panel was performed. Patients were classified as ‘probable’, ‘possible’ or ‘unlikely’ of having a pneumococcal pneumonia.

**Results:**

A sensitivity of 71.4% and specificity of 89.6% were found, corresponding to a negative predictive value and positive predictive value of 97.6% and 34.2%, respectively, when considering ‘probable’ versus ‘possible/unlikely’. In the PCR-positive cases we found a statistically significant difference in semi-quantitative Ct values between the ‘probable’ and the ‘possible/unlikely’ groups.

**Conclusions:**

We conclude that a negative qPCR for the *lytA* gene in a respiratory sample is highly predictive of a negative *S. pneumoniae* culture and is possibly sufficient to exclude *S. pneumoniae* as a causative agent. Respiratory pneumococcal PCR has a high negative predictive value for pneumococcal disease but the positive predictive value is low.

## Introduction

*Streptococcus pneumoniae* had already been recognized in the early years of clinical microbiology, when it was accidently discovered by Louis Pasteur and his colleagues, Chamberland and Roux, in the saliva of a child with ‘rage’ in 1881, during their search for the aetiological agent of rabies.^1^ Not long thereafter, the pathogen, although not recognized then as an organism belonging to the genus *Streptococcus*, was identified in numerous infectious diseases, from arthritis to meningitis, from abscesses to endocarditis, but especially as the main causative agent of bacterial pneumonia.^2^ In the 1940s, when case-fatality rates of bacterial pneumonia were still high, it was estimated that the pneumococci comprised 80% of cases of lobar pneumonia and 66% of cases of bronchopneumonia.^3^ In those years, it was already known that a high number of healthy people could carry one to several pneumococcal serotypes in the upper respiratory tract, making it difficult to assess the relevancy of a positive respiratory culture.^4^ With the start of the MALDI-TOF era of bacterial identification, technical challenges with this important pathogen persisted, as this revolutionary technology could not successfully distinguish *S. pneumoniae* from its close relatives (e.g. *Streptococcus mitis* group).^5^ This paved the way for the use of molecular techniques as fast and accurate means of identification, although the high sensitivity of molecular techniques in detecting the presence of *S. pneumoniae* makes the distinction between a carrier or its presens as the disease-causing agent all the more difficult.^6–8^ Different molecular targets have been studied to find a target that can most accurately identify virulent *S. pneumoniae* and differentiate it from non-pathogenic colonization. The autolysin *N*-acetylmuramoyl-L-alanine amidase, which is encoded by the *lytA* gene, plays an important role in pneumococcal pathogenesis and has already showed promising results as a target gene for the molecular diagnosis of pneumococcal pneumonia.^9^ Although false positives can occur due to the possible expression of the *lytA* gene in other streptococci, it is a frequently used component in respiratory multiplex PCR panels.^10–12^ Here, we present data on the meaning and utility of *lytA* gene detection of *S. pneumoniae* in a respiratory multiplex quantitative PCR (qPCR) panel for patients presenting with pneumonia.

## Materials and methods

A list of all patients admitted to our hospital (a 1000-bed community hospital) registered with a diagnosis of pneumonia, as diagnosed by the treating physician, from 1 July 2019 until 1 March 2022, was requested from the business intelligence department. Patients in whom pneumococcal PCR was not performed (as a component of a highly multiplexed respiratory qPCR panel) were excluded, as were children younger than 13 years, as they are known to be frequently colonized with *S. pneumoniae.*^4^ From included patients, pneumococcal urinary antigen test, respiratory and blood cultures, and remaining results from the respiratory PCR panel were retrieved.

**Figure 1.**
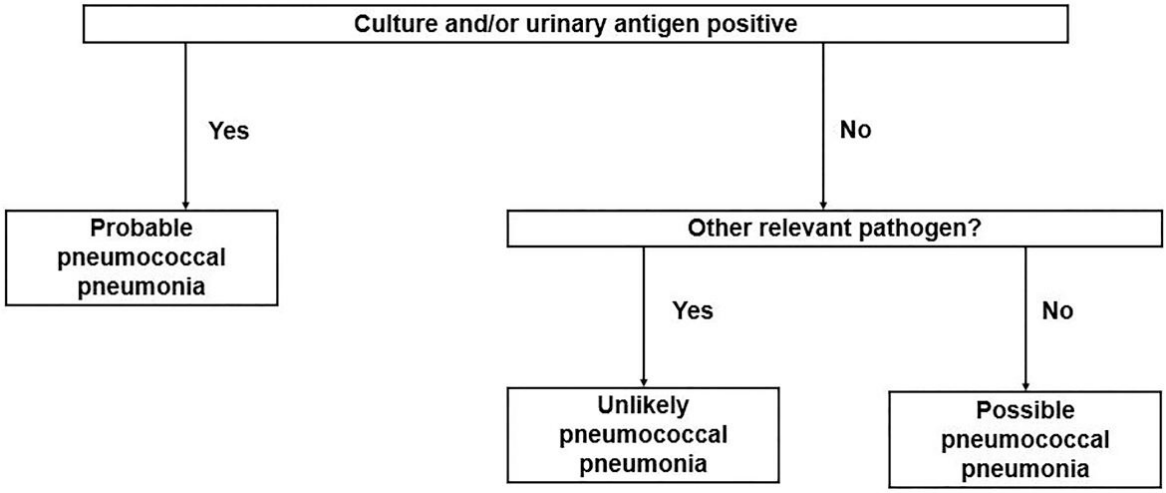
Probability of pneumococcal pneumonia categorization flowchart.

The *lytA* gene was targeted for the detection of *S. pneumoniae* as a component of a respiratory multiplex PCR panel. The PCR test was based on the method described by Gadsby *et al.*^12^ In order to increase the melting temprature of the probe, one extra nucleotide was added to the probe: Fwd_SP: 5′-ACGCAATCTAGCAGATGAAGCA-3′; Rev_SP: 5′-TCGTGCGTTTTAATTCCAGCT-3′; Probe_SP: 5′-TAMRA-TGCCGAAAACGCTTGATACAGGGAGT-BHQ2-3′. Phocine herpesvirus was added to the samples prior to extraction and amplification, and served as an internal control.^13^ PCR reactions were run on a Quantstudio-7 cycler (Thermo Fisher Scientific) in a total volume of 25 µL, using 6.25 µL of TaqMan Fast Virus master mix (Thermo Fisher Scientific) and a final oligonucleotide concentration of 300 nM for primers and 400 nM for the probe. The cycle parameters were as follows: 50°C for 5 min, 95°C for 20 s, followed by 45 cycles of 95°C for 3 s and 60°C for 30 s. The probability of pneumococcal respiratory disease for each hospitalization was determined according to the following methodology: ‘Probable’—a positive pneumococcal urinary antigen test or a positive pneumococcal culture (blood culture, bronchoalveolar lavage fluid, bronchus aspirate, rich growth in an endotracheal aspirate in the absence of other respiratory pathogens or a rich growth in sputum in the absence of other respiratory pathogens). ‘Unlikely’—no relevant growth of *S. pneumoniae* in culture and a negative pneumococcal urinary antigen test (if performed) and another relevant respiratory pathogen was identified. ‘Possible’—all others not categorized as ‘unlikely’ or ‘probable’ (Figure 1). Sputum samples underwent microscopic evaluation for quality, and those deemed to be of poor quality were excluded from further analysis.

Considered as relevant respiratory pathogens in culture were *Haemophilus influenzae*, *Moraxella catarrhalis*, *Klebsiella pneumoniae* and *Pseudomonas aeruginosa* when present in sufficient quantity according to the specimen type (confer *S. pneumoniae* in culture for ‘probable’ classification). Culture was not performed on nasopharyngeal swabs. Relevant respiratory pathogens detected with the multiplex qPCR panel were evaluated case by case according to the detected agent, the specimen type and the measured Ct value. Bronchoalveolar lavage fluid and bronchus aspirates were considered as deep (lower respiratory tract) specimens. Sensitivity, specificity, negative predictive value (NPV) and positive predictive value (PPV) were calculated in Excel. Pearson standardized residuals and chi-squared test were calculated in R. Figures were produced in R.

### Ethics

This study was performed in line with the principles of the Declaration of Helsinki and approved by the hospital ethics committee (approval reference number 220805RETRO). Given the retrospective nature of the study, it was determined that informed consent was not necessary.

## Results

**Figure 2.**
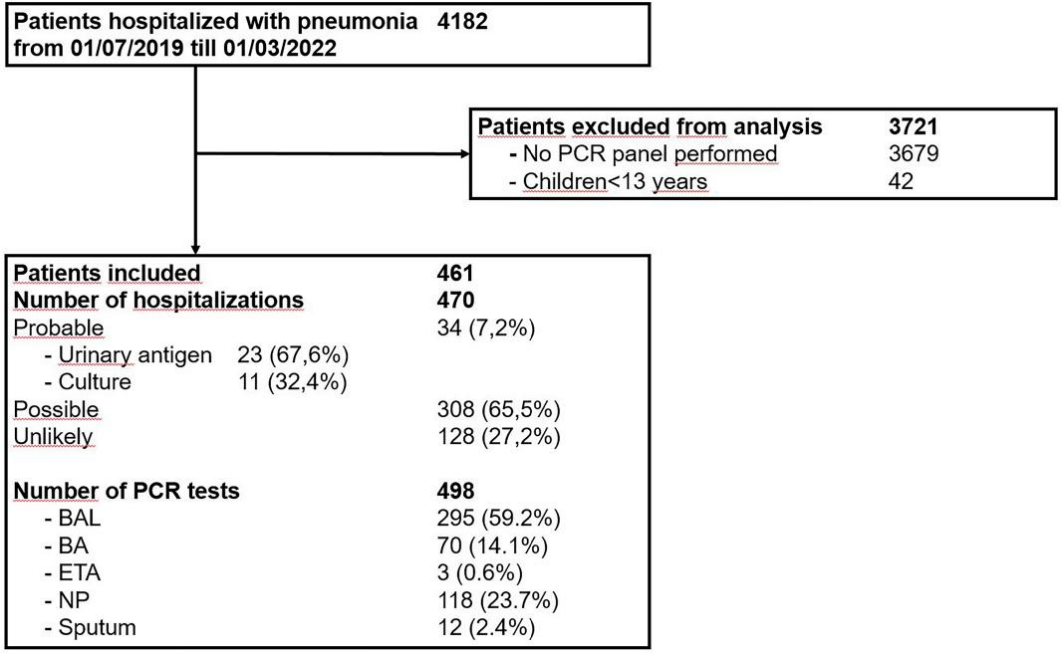
Inclusion flowchart and distribution among the probability of pneumococcal pneumonia categories. BA, bronchial aspirate; BAL, bronchoalveolar lavage fluid; ETA, endotracheal aspirate; NP, nasopharyngeal swab.

During the selected time period, 4182 patients were registered with pneumonia. Of these, 3721 (89.0%) were excluded from analysis: 3679 patients were excluded due to the absence of a respiratory PCR multiplex result (88.0%, mean age 71 years) and 42 patients (1%) were younger than 13 years and therefore also excluded from the cohort. Four hundred and sixty-one unique patients were withheld, accounting for 470 different hospitalizations and 498 different respiratory specimens (Figure 2). Of the 461 patients included, the mean age was 64 years (mean deviation of 15 years).

**Figure 3.**
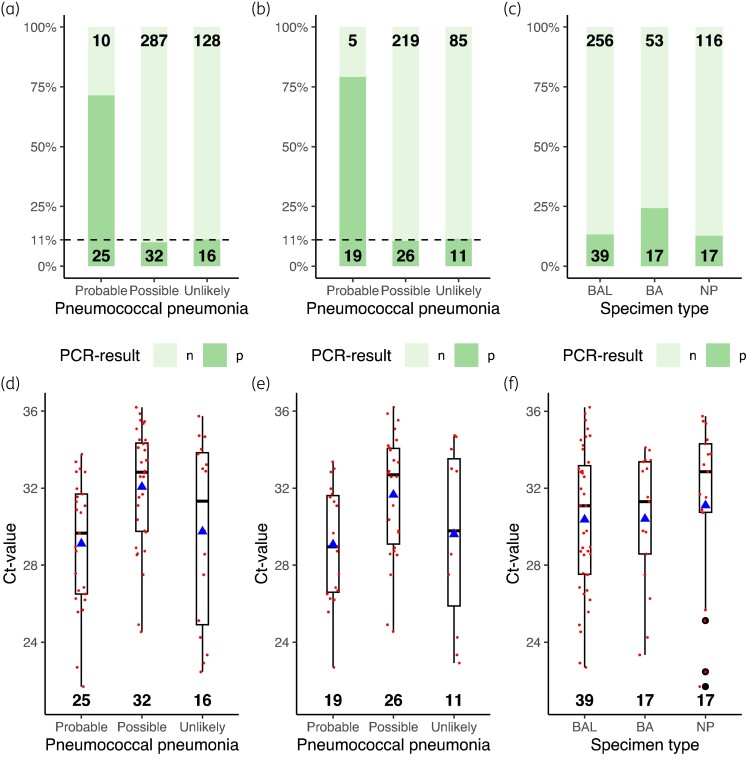
Results of the *lytA*-PCR. (a) Distribution of qualitative results according to the probability of having a pneumococcal pneumonia. (b) Distribution of qualitative results in deep specimens (BAL and BA) according to the probability of having a pneumococcal pneumonia. (c) Distribution of qualitative results according to the specimen type. (d) Distribution of semi-quantitative qPCR results (Ct value) according to the probability of having a pneumococcal pneumonia. (e) Distribution of semi-quantitative qPCR results (Ct value) in deep specimens according to the probability of having a pneumococcal pneumonia. (f) Distribution of semi-quantitative qPCR results (Ct value) according to the specimen type. Box and whisker plots parameters: 0, 0.25, 0.5, 0.75 and 1 quantiles. BA, bronchial aspirate; BAL, bronchoalveolar lavage fluid; Ct, cycle threshold; n, negative; NP, nasopharyngeal swab; p, positive; qPCR, quantitative polymerase chain reaction; filled triangle, Ct value mean.

The *lytA* gene was detected in 10.4% of specimens (*n* = 48) from patients who were not classified as having a probable pneumococcal respiratory infection (*n* = 436). In 71.4% (*n* = 25) of those classified with a probable pneumococcal respiratory infection (*n* = 35), the *lytA* gene could be detected (Table S1, available as Supplementary data at *JAC-AMR* Online). All patients classified as having a probable pneumococcal respiratory infection without a positive pneumococcal PCR (*n* = 10) were classified as ‘probable’ on the basis of a positive urinary antigen test without a positive culture. Five of these PCR-negative, but *S. pneumoniae*-probable cases (50%), were tested with nasopharyngeal swabs. No difference in median Ct values could be observed for patients with a higher likelihood of having a true pneumococcal pneumonia (‘probable’ versus ‘possible/unlikely’) nor in the specimen type used (chi-squared test) (Figure 3). When comparing the semi-quantitative Ct values between the ‘probable’ versus ‘possible/unlikely’ group (Mann–Whitney *U* test) (as we cannot assume a normal distribution according to the Shapiro–Wilk test), a statistically significant difference could be observed (*P* < 0.01). No difference was observed in positivity rate among the specimen types (chi-squared test). More nasopharyngeal swabs were used in the ‘unlikely’ category (1.54 Pearson standardized residuals) and fewer nasopharyngeal swabs were used in the ‘probable’ category (−1.2 Pearson standardized residuals), although this difference in distribution was not statistically significant (*P* value chi-squared test = 0.06).

Calculated sensitivity and specificity for all specimen types was 71.4% and 89.6%, respectively, when considering the ‘probable’ category against the ‘possible/unlikely’ category. This corresponds to an NPV and PPV of 97.6% and 34.2%, respectively. When taking only deep specimens into account, sensitivity would increase to 81.1% and specificity would decrease to 89%. This coincides with an increase in the NPV to 98.7% and a decrease of the PPV to 32.7%.

## Discussion

Results are concordant with a previous evaluation of the *lytA* gene qPCR for the diagnosis of pneumococcal pneumonia using sputum or nasopharyngeal swab in the elderly, in that no useful conclusion can be made using semi-quantitative Ct values for true pneumococcal disease.^14^ Although it may not be clinically applicable for establishing a clear-cut threshold, we did observe a statistically significant difference in the semi-quantitative Ct value between the ‘probable’ group and the ‘possible/unlikely’ group. We do consider the absence of detection a good marker for the exclusion of pneumococcal disease. In our cohort, the *lytA* gene was not detected in 10 patients in whom pneumococcal pneumonia was considered probable. The categorization of all these patients into the ‘probable’ group was based on a positive urinary antigen test. In none of these patients was *S. pneumoniae* cultured from the respiratory sample. Possibly, some of these patients had a positive pneumococcal urinary antigen test, without having a true pneumococcal pneumonia, as cross-reactivity with other streptococci has been described for the urinary antigen test.^15^ Half of the PCR-negative probable cases were tested with nasopharyngeal swabs. Therefore, it is important to take into account the potential lower sensitivity associated with these sample types. Considering an NPV of 97%–99%, absence of *lytA* gene detection is a useful marker in the diagnostic work-up of patients presenting with pneumonia.

There are several limitations in our study. First, despite the initial large patient cohort, only a few patients with a probable pneumococcal pneumonia could be included. Second, the retrospective character, which allowed us to examine a bigger cohort, made it impossible to standardize the molecular tests and measure a quantitative log-scale. This reduces the generalizability of our results. A prospective study would also allow a uniform diagnostic work-up among patients presenting with pneumonia, allow a better segregation in probability of a pneumococcal pneumonia and would make it possible to verify positive urinary antigen tests of precarious uncertainty. It should also be noted that although we used a positive urinary antigen test and/or positive culture to define probable cases, these are not gold standards for diagnosing a pneumococcal pneumonia. As Kakiuchi *et al.*^16^ pointed out, the estimated sensitivity and specificity of qPCR for *lytA* is higher than those of the urinary antigen and culture when using Bayesian latent class models.

One could question the relevance of a pneumococcal PCR in a multiplex PCR panel because the result would not directly alter antibiotic management. When the pneumococcal PCR is positive the PPV of the test is too low to tailor the empirical antibiotic to a narrow-spectrum penicillin. When the PCR is negative the high NPV probably excludes pneumococcal disease but empirical antibiotics would have to be continued because other pathogens (*H. influenzae*, *M. catarrhalis*, etc.) are not excluded. Nevertheless a pneumococcal PCR is often included in respiratory multiplex PCR panels, both commercial and in-house. We believe the main strength of our research is clarification of the meaning of the pneumococcal PCR result, negative or positive, which is a very common question in most clinical laboratories.

We can conclude that a negative qPCR for the *lytA* gene is highly predictive of a negative *S. pneumoniae* culture in a respiratory sample and is possibly sufficient to exclude *S. pneumoniae* as a causative agent.

## Supplementary Material

dlad115_Supplementary_Data

## Data Availability

The dataset analysed for the current study is not publicly available due to GDPR but is available from the corresponding author on reasonable request.

## Supplementary data

Table S1 is available as Supplementary data at *JAC-AMR* Online.
