# Supplementary material for: Value of pneumococcal PCR in respiratory samples for exclusion of pneumococcal pneumonia
Source: JAC Antimicrob Resist. 2023 Nov 2;5(6):dlad115. doi: 10.1093/jacamr/dlad115 (PMC10629468; doi:10.1093/jacamr/dlad115)
Supplement: dlad115_Supplementary_Data [file dlad115_supplementary_data.docx]

| Likelihood of disaese  LytA-gene detection | Probable | Possible / unlikely |
| --- | --- | --- |
| + | 25 | 48 |
| - | 10 | 415 |
| Total | 35 | 463 |

Supplementary table 1. Distribution of positive and negative LytA-gene PCR test result among probable cases and possible or unlikely cases.
